# Adapting HIV patient and program monitoring tools for chronic non-communicable diseases in Ethiopia

**DOI:** 10.1186/s12992-016-0163-y

**Published:** 2016-06-02

**Authors:** Mekitew Letebo, Fassil Shiferaw

**Affiliations:** Clinton Health Access Initiative, Addis Ababa, Ethiopia; World Health Organization, Addis Ababa, Ethiopia

**Keywords:** HIV, NCD, Monitoring, Evaluation, Adaptation, Ethiopia

## Abstract

**Background:**

Chronic non-communicable diseases (NCDs) have become a huge public health concern in developing countries. Many resource-poor countries facing this growing epidemic, however, lack systems for an organized and comprehensive response to NCDs. Lack of NCD national policy, strategies, treatment guidelines and surveillance and monitoring systems are features of health systems in many developing countries. Successfully responding to the problem requires a number of actions by the countries, including developing context-appropriate chronic care models and programs and standardization of patient and program monitoring tools.

**Methods:**

In this cross-sectional qualitative study we assessed existing monitoring and evaluation (M&E) tools used for NCD services in Ethiopia. Since HIV care and treatment program is the only large-scale chronic care program in the country, we explored the M&E tools being used in the program and analyzed how these tools might be adapted to support NCD services in the country. Document review and in-depth interviews were the main data collection methods used. The interviews were held with health workers and staff involved in data management purposively selected from four health facilities with high HIV and NCD patient load. Thematic analysis was employed to make sense of the data.

**Results:**

Our findings indicate the apparent lack of information systems for NCD services, including the absence of standardized patient and program monitoring tools to support the services. We identified several HIV care and treatment patient and program monitoring tools currently being used to facilitate intake process, enrolment, follow up, cohort monitoring, appointment keeping, analysis and reporting. Analysis of how each tool being used for HIV patient and program monitoring can be adapted for supporting NCD services is presented.

**Conclusion:**

Given the similarity between HIV care and treatment and NCD services and the huge investment already made to implement standardized tools for HIV care and treatment program, adaptation and use of HIV patient and program monitoring tools for NCD services can improve NCD response in Ethiopia through structuring services, standardizing patient care and treatment, supporting evidence-based planning and providing information on effectiveness of interventions.

## Background

Chronic non-communicable diseases (NCDs), particularly cardiovascular diseases, diabetes, cancer and chronic respiratory diseases are the leading causes of mortality worldwide with an estimated 38 million deaths each year [1]. According to the World Health Organization (WHO), the contribution of NCDs to years of life lost (YLL) has gone up globally from 38 % in 2000 to 47 % in 2012 [2]. Besides, the burden of these diseases is going to grow in the coming years, with Africa projected to have the greatest increase [3]. It is, however, worth emphasizing that NCDs have already become a huge public health problem in many African countries, including Ethiopia where NCDs were responsible for an estimated 30 % of all deaths in 2014 [1].

Lack of organized and comprehensive response has been the feature of NCD response in many developing countries, including Ethiopia. Systems for NCD response such as national policy, strategies, treatment guidelines and surveillance and monitoring systems are either non-existent or at an early stage in many African countries [1]. NCD care available to patients in these countries within the context of primary health care (PHC) is characterized by problems in access, equity and responsiveness [4]. Besides, the services lack strong systems for monitoring and surveillance, resulting in lack of data to inform policy formulation and designing strategies and programs [5]. Having such systems in place is vital for successful control of NCDs and there is already evidence from resource-poor countries on how data on burden of NCDs and barriers to NCD care could facilitate implementation of NCD programs [6]. Overall, the need to improve health system response to NCDs, especially in the context of PHC, is being emphasized and a variety of frameworks and strategies are being proposed [7, 8].

In Ethiopia, national response to NCDs is in its infancy stage with very little done in the way of policy formulation, prevention and control strategies and program design for effective NCD response. Small-scale studies on NCD burden of disease and risk factors started to appear since 2008, and the country embarked on drafting national strategic framework in 2010. This was followed by development of a national action plan for 2014–2016 in 2014. Policy and guidelines for prevention and treatment of NCDs are yet to be developed.

The need to re-orient the health system in Ethiopia is clear as the system is mainly designed for the provision of acute and episodic health care and experience with chronic care has been limited except for HIV care and treatment program. It is widely believed that scale up of antiretroviral therapy (ART) over the past decade has helped the country obtain valuable experience providing a continuum of care with relatively limited resources and facilities in PHC setting. A number of factors have contributed to the relative success with this approach, including the adoption of WHO’s Integrated Management of Adolescent and Adult Illness (IMAI) approach, which has provided modules covering wide-ranging issues, including acute care, chronic HIV care including ARV therapy, palliative care and general principles of good chronic care. The latter is meant to support the health systems to transition from acute to chronic care [9]. In line with the IMAI approach, Ethiopia standardized and integrated the delivery of comprehensive HIV service into the existing health system, ultimately scaling up prevention, care and treatment services throughout the country starting from the year 2005 [10]. Among the key features of the approach were standardization and simplification of laboratory testing and treatment, task shifting, and the use of the standardized patient and program monitoring and evaluation (M&E) system. Like many other resource-limited settings, as a result of ART scale up, Ethiopian health care system is now considered to be better positioned to plan and implement chronic care services such as for NCDs.

However, compared to HIV, provision of comprehensive services for NCDs poses a number of challenges to resource-poor countries due to the diverse disease types with differing risk factors, clinical presentations and treatment modalities; the high burden of disease; presence of competing priorities; weak health systems and inadequate partnerships and resource mobilization. Given these, prevention and control of NCDs appear to be different and perhaps more complex than HIV/AIDS. However, a focused programmatic approach with emphasis on standardization and integration of treatment for selected high-burden NCDs, such as cardiovascular diseases, selected cancers and diabetes, and reduction of the four major risk factors to NCDs, i.e., tobacco use, physical inactivity, harmful use of alcohol and salt/sodium intake, will help reduce premature mortality from NCDs. In doing so, although replicating the relative success of HIV program may not be straightforward, the many lessons and capacities built as a result of ART scale up in resource-limited settings would still be useful in informing the designing and implementation of NCD prevention and control strategies. In fact, many organizations, agencies, and scholars have long called for leveraging the HIV/AIDS program for prevention and control of NCDs, and some have started applying aspects of HIV care and treatment approaches to selected NCDs in resource-poor countries.

While most of the calls for building upon the success of HIV program were made in relation to the health care delivery models [11], one area with growing interest is the need for adapting HIV services’ monitoring and evaluation systems, approaches and tools to NCD care and treatment. There have also been actual applications of aspects of HIV patient monitoring systems for NCD care such as the use of cohort monitoring approaches for NCD patients [12–14]. There are cases where HIV patient monitoring tools are adapted and implemented for cohort monitoring of those with hypertension and diabetes with illustrations on how such systems might improve patient outcomes and program monitoring [15, 16]. On the other hand, there are also those who suggested adaptation of monitoring systems for other chronic diseases such as the WHO “DOTS” monitoring framework for tuberculosis [17, 18], an approach applied for diabetes care in Malawi [19]. The latter is a clear indication that given the diversity of diseases and conditions within NCDs and the complexity surrounding the prevention and control efforts, exploring models other than HIV care may also be needed for successful NCD response.

The aim of this study is to provide a comprehensive link between HIV services’ M&E system and the existing NCD services M&E system/tool and to discuss how NCD care and treatment service delivery and patient outcomes can be supported through adaptation and use of HIV services’ M&E tools. The study also aims at providing the practicalities surrounding this process, including the possible challenges in adapting HIV patient and program monitoring tools for NCDs.

## Methods

M&E tools currently being used in HIV care and treatment program and existing NCD patient and program monitoring tools have been examined through document review, observations and in-depth interviews. The investigators visited two hospitals, and two health centers purposively selected based facility type and having high HIV as well as NCD patient load. One of the hospitals has the highest number of patients on HIV care and treatment in the country. In each health facility, data clerk, ART provider, Health Management Information Systems (HMIS) focal person, and ART focal person were interviewed. A total of fourteen participants were interviewed. In the health centers the ART focal persons were also ART providers. All health workers interviewed had experience with both HIV care and treatment services and management of patients with NCDs.

Participants were asked to identify all HIV patients and program monitoring tools currently being used in the facilities. They were then asked to provide information on each tool, including the purpose, how it is used, user groups and level of use. In addition, participants were asked if they have similar tools for managing NCD patients and programs with specific examples of hypertension and diabetes used to facilitate discussion. When their response is “yes” they were asked to make comparisons between the tools and when it is “No” they were asked if they would like to have similar tools for NCDs and the reasons. Data clerks and HMIS focal persons were asked about the potential role of these tools from perspective of data management while health workers were asked to discuss the role of these tools in patient management and linking them to treatment outcomes. ART focal persons were also asked about the possible role of similar tools for program management.

Interview sessions lasted an average of 25 min, and hand-written notes were taken. Besides, key strategic documents as well as all paper and electronic data collection, aggregation, processing, presentation and reporting tools that exist for both HIV care and treatment, and NCD services were critically reviewed by the authors. Data were analyzed manually using thematic content analysis. All NCD and HIV services’ M&E tools and field notes from in-depth interviews were thoroughly reviewed for themes on how and why a particular tool is being used in each program, gaps that exist in NCD services’ M&E systems in terms of addressing patient and program needs, and how the gaps could be addressed if HIV patient and program monitoring tools were to be adapted and used for NCD services.

Ethical clearance was obtained from the local research and publication committee from respective health facilities. Informed consent was also secured from the participants. Confidentiality of personal information was maintained throughout the study period, and the study was done in line with Helsinki Declaration.

## Results

### HIV care & treatment M&E system in Ethiopia

Ethiopia’s HIV patient care and treatment monitoring system is designed and implemented based on WHO guidelines for HIV patient monitoring [20]. Generic tools provided by WHO were adapted by Federal ministry of health (FMOH) and implemented in every health facility providing HIV care and treatment services from the outset. ART scale up was substantially aided by the use of such tools in a standardized manner which helped to organize patient care and allowed systematic program monitoring. The generic tools adapted for use in the country were intake and follow up forms, Pre-ART and ART registers, monthly reporting forms, and cohort reports. In addition, a variety of tools which are based on the adapted generic tools and are believed to strengthen the M&E system further were designed and introduced by implementing/developmental partners supporting HIV care and treatment in the country. These include various paper-based tools and patient level databases. Clear recognition of M&E as an essential element to the program and sustained support from government and other stakeholders were among the key features of HIV/AIDS M&E system in Ethiopia.

Our interviews with data clerks, health workers, and ART focal persons in the four health facilities we visited reveal that at least 11 major data collection, aggregation, analysis, presentation and use tools are currently available in ART clinics. Of these, six are the standardized tools introduced by FMOH while the rest are developed and implemented by partner organizations with approval from the ministry of health. The role of each tool in HIV care and treatment and their potential place for NCD services as suggested by health workers and data managers are presented below. In addition, participants’ response and our review of whether similar tools are available for NCD services or not are outlined. For the tools that are available, the similarities and differences between the corresponding tools are assessed from the perspective of patient and program monitoring. Our analysis is limited to the M&E tools. Other aspects of the M&E system are not addressed in this paper. Although a given tool can have more than one use, to facilitate the presentation of findings and discussion the tools are categorized as data collection; aggregation; processing, analysis and presentation; and reporting and self-assessment tools based on their predominant role.

### Data collection tools

Our assessment identified two main tools used for collecting patient level data in ART clinics: intake and follow up forms. Intake forms are filled out only once at enrollment while follow up forms are updated each time patients visit the clinic. The role of these tools in HIV care and treatment and their potential use for NCD services as suggested by participants are summarized in Table 1.

**Table 1:** Potential role of HIV care & treatment intake and follow up forms for NCD services

Tool	Role in HIV care and treatment	Suggested uses for NCD services
Intake forms	Facilitate patient enrolment into HIV care	Help establish appropriate patient-health worker relationships
	Systematically identify barriers and facilitators to adherence to ART	Identify potential barriers to compliance with treatment
	Collect baseline information on comorbidities, opportunistic infections(OIs), support system, disease stage	Identify comorbidities, complications, disease stage, and support system
	Aid adherence and risk behavior counselling	Organize counseling on lifestyle modification such as diet, exercise, and other issues
	Help in treatment planning	Facilitates treatment planning
Follow up forms	Provides information on pattern of follow up	Ensuring regular follow up
	Type and duration of treatment	Treatment type and compliance monitoring
	Assessment of need for change in treatment	Assessment of whether treatment is working or not and need for change
	Early detection and management of drug side effects	Can help structure lifestyle changes counselling
	Organized screening and monitoring of common complications (early detection)	Facilitate systematic side effects monitoring and management
	Facilitates and reminds important lab testing	Facilitates systematic clinical and laboratory screening of complications and other risk factors
	Helps provide other care in integrated manner (family planning, STI)	Integrating service packages
	Guides objective and frequent assessment of adherence to treatment and	Organizes treatment and care history and ensures continuity of care in case of staff rotations and turnover
	Facilitates planning for next appointment	Joint planning of next appointment and setting treatment targets
	Provide data for research and cohort monitoring	Support research on NCDs
		Facilitates referral to various services

The health workers and data managers did, however, have concerns about the use of these tools for NCD services. Some of the respondents described intake forms as complex and time consuming while a few health workers explained the use of such tools could complicate data collection. Others stated that ensuring sustained supply of these tools, which, for ART program, was effectively handled through printing and distribution by partner organizations, could be challenging.

Despite these concerns, however, the use of these tools for NCDs has been recommended by all our participants though with reservation in some cases. Two of the physicians we interviewed, for example, stated that the tools may not have the exact same impact they had on HIV care and treatment due to the very diverse NCD patient population. However, they emphasized, any attempt to provide organized NCD service at PHC level without the use of similar tools is unlikely to yield any results.

### Data aggregation tools

The main data aggregation tools used in ART clinics are pre ART and ART registers. These registers are used to record selected key information on individual patients needed for cohort analysis and reporting. These registers facilitate compilation of monthly and cohort reports, which are shared to health managers for higher level decision making on resource allocation, including human resource, drugs and laboratory. These data feed all the way up to the national and global level and provide the much-needed data on access for treatment and success of treatment programs.

Our review showed that no such registers specifically designed for aggregating data on NCDs exist in health facilities in Ethiopia. Data on NCDs such as hypertension and diabetes is aggregated as part of outpatient and inpatient registers which also contain data from a number of other conditions. No link can be found between various previous visits making any meaningful interpretation and analysis of data from individual patients as well as groups impossible. The only details that can be found from these registers are the diagnosis and treatment given on that particular visit.

According to our participants, registers which are specifically designed for NCDs which help capture key diagnosis, treatment, screening and counseling data on individual and groups of patients would help the services a great deal. All the health workers we interviewed recommend the use of similar tools for NCD services, although some of them stated the additional time required to fill out the registers could take some time from their busy schedule.

### Data processing, analysis and presentation tools

The main tool we identified in this regard is the patient-level ART database. All the four health facilities we visited had such databases and were actively using them for a variety of purposes. According to the participants, the databases are being used for storing, retrieving and analyzing data. They also stated that, although with some errors, the databases generated all the required reports. The databases also facilitated the compilation of ad-hoc reports as they make data retrieval and summarizing easier. Data clerks also stated that they used the database to keep appointments. Specifically, clinic attendance is checked and patients who missed appointments are identified using the database. The database also provides a detailed address of the patients to help initiate tracing. The databases have also been occasionally used by data clerks to replace lost patient folders as they can generate exact copies of the paper-based intake and follow-up forms up until the last date of follow-up. This, according to the data clerks, is vital in terms of preventing loss of valuable data needed for patient care and program management. Similarly, databases have allowed backing up of the whole patient level and program data at multiple sites ensuring no data are permanently lost. Another specific and practical use of the databases identified by the data clerks was enabling searching patients’ card numbers when patients visit the facilities without their small card, which provides the details needed to get their treatment folders. This, they believe, has helped patients from returning home without receiving the care and treatment they seek. Other benefits of the databases identified by the participants include data quality assurance through in-built validation tools, obtaining summaries on patient outcomes to support decision making and availability of electronic data for research purpose. All health workers suggested that all these applications could be useful for managing patients with NCDs such as those with hypertension and diabetes.

### Reporting and self-assessment tools

Table 2 provides details on the three main tools currently being used in ART clinics, their role in HIV care and treatment and suggested uses in NCD patient and program monitoring.

**Table 2:** Potential role of HIV care & treatment monthly and cohort reports and self-assessment tools for NCD services

Tool	Role in HIV care and treatment	Suggested uses for NCD services
Monthly reports	Provide age and sex disaggregated facility, district, regional and national level data on enrolment, number on treatment, and drugs being used	Number with a given NCD with age and sex breakdown at each level, medications being used
	Help quantification of disease burden and facilitates resource planning at each level	Quantification of disease burden and risk level for planning and program design
	Provides data on access for treatment at local level and beyond	Data on access for care and treatment
Cohort charts/reports	Treatment outcome of groups	Treatment outcome of groups
	Retention on treatment	Patient retention on care and treatment
	Survival analysis	Survival analysis for different NCDs and different treatments
	Changes in clinical, functional and immunologic outcomes	Monitoring quality of NCD services
	Monitoring service quality and risk of drug resistance	
Self-assessment forms	Self-evaluation based on service specific key performance indicators	Self-evaluation for continuous quality improvement

Our review revealed that NCDs are reported together with other diseases through quarterly reports. It is difficult to extract data pertaining to specific diseases for quantification of disease burden and resource planning. No cohort and self-assessment forms exist for NCD services.

### Other tools

Two other tools we identified at the ART clinics which are being used to support patient management are the appointment calendar and adherence log book. These tools are primarily used for tracking attendance, listing those who missed appointments, document actions taken by the facility (usually adherence supporters) including tracing, and outcomes of tracing. Based on these logbooks, indicators on adherence are calculated using self-assessment forms. Participants stated that similar tools could be used to support management of NCD patients emphasizing the fact that effective management of most NCDs also requires a high degree of compliance to medication. However, they emphasized that the services may need to be organized in a manner similar to HIV care and treatment for successful use of such tools.

## Discussion

Chronic NCDs have emerged as a significant public health challenge in developing countries like Ethiopia where the burden is high. The health systems in these countries seem ill-equipped to adequately respond to the challenge [21, 22] with NCD programs generally poorly funded, organized, and monitored [5, 21, 23, 24].

There is clearly a lack of experience providing chronic care in many resource-poor countries where the huge burden of communicable diseases has precedence over NCDs. The exception to this is the experience in the HIV care and treatment program. Although replicating the kind of response mobilized for HIV response is extremely difficult, many argue that leveraging the chronic care experience and infrastructure developed for chronic HIV care and adaptation of its programmatic aspects would be a practical and sensible way to respond to the NCDs in poor countries [11–14]. In fact, there are few developing country contexts where adapting programs for NCDs has been tried. Attempts by the Zambian Ministry of Health to adapt aspects of HIV program for NCDs [25] and the use of HIV clinic approach to enhance diabetes care in a hospital in Ethiopia [14] are among the examples. There is also a reported experience on a successful provision of chronic care for NCDs within a PHC context in Western Ethiopia [26]. However, large-scale provision of NCD care and treatment in a PHC setting with viable chronic care models is still a long way in most developing countries.

One important aspect of NCD programming often overlooked is its monitoring, evaluation and surveillance system [4, 24]. M&E, surveillance and research have been among the key contributors to the success of HIV programs in developing countries. A strong M&E system based on standardized and simplified patient and program monitoring tools has helped resource-poor countries like Ethiopia successfully scale-up ART services up to the remotest PHC settings. Paying attention to the M&E system of NCD programs and possible adaptation and use of HIV services’ M&E system could, therefore, improve NCD services in such countries. So far, there is limited experience applying aspects of HIV services’ M&E systems for management of NCD patients and programs. The use of cohort monitoring approach for hypertension [15] and diabetes [16] is among the few limited illustrations of such applications. Given the need for huge human and capital resources required to adopt electronic health records and decision support systems currently widely used for managing NCD patients and services in developed countries, building on the already established HIV care M&E infrastructure would be the most reasonable step to take.

HIV care and treatment in Ethiopia have been supported by a number of powerful M&E tools, which played key role in service delivery and program management. Our review showed that no such tools exist for NCD services at the moment. Given the similarity in several aspects of NCD services and HIV care and treatment, many of these tools can be successfully adapted for use in NCD services. Intake forms can facilitate and formalize enrolment of patients with NCD into chronic care while at the same time helping the health care providers systematically look for complications, comorbidity, barriers to adherence to treatment, and baseline values on different clinical and laboratory parameters for comparison during subsequent follow ups. The revised shorter versions of intake forms currently being developed for HIV care can be a starting point for adapting for NCD services. Follow up forms would clearly be vital for providing appropriate continuity care for patients with NCDs. As with HIV, follow up forms would help ensure patients are appropriately screened for complications, medication side effects and poor compliance to treatment during each encounter. Well-designed follow up forms can help organize care givers’ clinical decision-making process through structured and chronological display of key clinical, laboratory and treatment compliance data which the usual orthodox method of documentation on a history sheet does not provide. These patient-level tools are particularly important to ensure the provision of minimum essential care and conduct of appropriate referral in PHC settings where there are very few specialists and general practitioners.

NCD registers can make data aggregation and report generation easier. This can help produce reports, which are vital in terms of providing key information to managers and decision-makers, including on disease burden, risk factors and access to treatment. Application of cohort monitoring approach and analysis using cohort charts tried in some settings for patients with diabetes and hypertension can also provide valuable information on treatment outcomes. Self-assessments are vital for any program as they provide an opportunity for self-evaluation and instituting corrective actions in a timely manner. Studies from parts of Ethiopia show that adherence to diabetes medications is very poor, and a lot of barriers exist [27]. Thus manual appointment keeping systems and adherence log books like the ones being used for HIV care and treatment can also benefit NCD services as they could help track attendance and ensure regular follow up. Adapting patient-level ART databases would substantially aid NCD service delivery. Electronic databases can store data and make retrieval of information easier. They can also be used to generate reports, track attendance, provide summaries on key program indicators, and improve the quality of health information collected. Furthermore, databases can support conduct of operational and clinical research to generate knowledge that would support continuous quality improvement efforts.

## Conclusions

Standardized and simplified M&E system would be central to successful provision and scale-up of NCD services in developing countries. Currently, very few M&E tools supporting NCD services exist in Ethiopia. There are, however, several paper and electronic patient level as well as aggregate M&E tools being used for HIV care and treatment services in the country. Carefully adapted, some of these M&E tools can help facilitate intake process, enrolment, follow up, cohort monitoring, appointment keeping, analysis and reporting of NCD services. Besides, the tools can help structure services, standardize care and treatment and support evidence-based planning. Thus, alongside developing context-appropriate NCD service delivery models and programs, the possibility of adapting HIV patient and program monitoring tools for NCD services should be assessed, especially if expansion of NCD services to PHC settings is to be considered. Although adapting and implementing such tools could place some demand on the country’s resources, it would be cheaper than developing tools from scratch.

Alongside adapting these tools, developing electronic health records for chronic diseases, preferably using open-source software, would be an important consideration to make. The two patient-level ART databases we observed in the health facilities we visited were not open-source and support was not readily available. Currently, a free and open source system called DHIS (District Health Information Systems) is one of the commonly used systems in developing countries. The system is highly configurable and has a strong global network of developers and support team called HISP (Health Information Systems Project). Moving to systems like DHIS could thus be the best option to take. In addition, given the current momentum towards using a variety of mHealth applications for improving management of NCDs and the hugely expanding mobile network and ownership in Ethiopia, the use of mHealth applications such as mAdherence tools and tools that support self-care of patients with chronic diseases should also be considered for its utility in NCD programs.
